# An exploratory study of pro-inflammatory cytokines in individuals with alcohol use disorder: MCP-1 and IL-8 associated with alcohol consumption, sleep quality, anxiety, depression, and liver biomarkers

**DOI:** 10.3389/fpsyt.2022.931280

**Published:** 2022-08-11

**Authors:** Narjis Kazmi, Gwenyth R. Wallen, Li Yang, Jenna Alkhatib, Melanie L. Schwandt, Dechun Feng, Bin Gao, Nancy Diazgranados, Vijay A. Ramchandani, Jennifer J. Barb

**Affiliations:** ^1^Translational Biobehavioral and Health Disparities Branch, National Institutes of Health, Clinical Center, Bethesda, MD, United States; ^2^Office of the Clinical Director, National Institute on Alcohol Abuse and Alcoholism, National Institutes of Health, Bethesda, MD, United States; ^3^Laboratory of Liver Diseases, Division of Intramural Clinical and Biological Research, National Institute on Alcohol Abuse and Alcoholism, National Institutes of Health, Bethesda, MD, United States; ^4^Human Psychopharmacology Laboratory, Division of Intramural Clinical and Biological Research, National Institute on Alcohol Abuse and Alcoholism, National Institutes of Health, Bethesda, MD, United States

**Keywords:** cytokine, sleep quality, PSQI, AUD, inflammation, liver, anxiety, depression

## Abstract

**Background:**

High levels of sleep disturbances reported among individuals with alcohol use disorder (AUD) can stimulate inflammatory gene expression, and in turn, may alter pro-inflammatory cytokines levels. We aimed to investigate associations between pro-inflammatory cytokine markers with subjective measures of sleep quality, psychological variables and alcohol consumption among individuals with AUD.

**Methods:**

This exploratory study is comprised of individuals with AUD (*n* = 50) and healthy volunteers (*n* = 14). Spearman correlation was used to investigate correlations between plasma cytokine levels and clinical variables of interest (liver and inflammatory markers, sleep quality, patient reported anxiety/depression scores, and presence of mood and/or anxiety disorders (DSM IV/5); and history of alcohol use variables.

**Results:**

The AUD group was significantly older, with poorer sleep quality, higher anxiety/depression scores, and higher average drinks per day as compared to controls. Within the AUD group, IL-8 and MCP-1 had positive significant correlations with sleep, anxiety, depression and drinking variables. Specifically, higher levels of MCP-1 were associated with poorer sleep (*p* = 0.004), higher scores of anxiety (*p* = 0.006) and depression (*p* < 0.001), and higher number of drinking days (*p* = 0.002), average drinks per day (*p* < 0.001), heavy drinking days (*p* < 0.001) and total number of drinks (*p* < 0.001). The multiple linear regression model for MCP-1 showed that after controlling for sleep status and heavy drinking days, older participants (*p* = 0.003) with more drinks per day (*p* = 0.016), and higher alkaline phosphatase level (*p* = 0.001) had higher MCP-1 level.

**Conclusion:**

This exploratory analysis revealed associations with cytokines MCP-1 and IL-8 and drinking consumption, sleep quality, and anxiety and depression in the AUD group. Furthermore, inflammatory and liver markers were highly correlated with certain pro-inflammatory cytokines in the AUD group suggesting a possible relationship between chronic alcohol use and inflammation. These associations may contribute to prolonged inflammatory responses and potentially higher risk of co-morbid chronic diseases.

## Introduction

Alcohol use disorder (AUD) is characterized by the impaired ability to stop or control alcohol use, even when patterns of drinking have negative social, occupational, and health consequences (1). According to the 2019 US National Survey of Drug Use and Health, AUD has an estimated prevalence of 5.6% in adults ages 18 and older (2). AUD can stem from several risk factors, some of which being an early onset of drinking, genetic influence, sleep related disorders, and the presence of mental health conditions (3–6). Moreover, due to the complex and bi-directional relationship between alcohol use and sleep-related disorders, high levels of sleep disturbances have also been documented as a common symptom in individuals with AUD (7).

**Figure 1:**
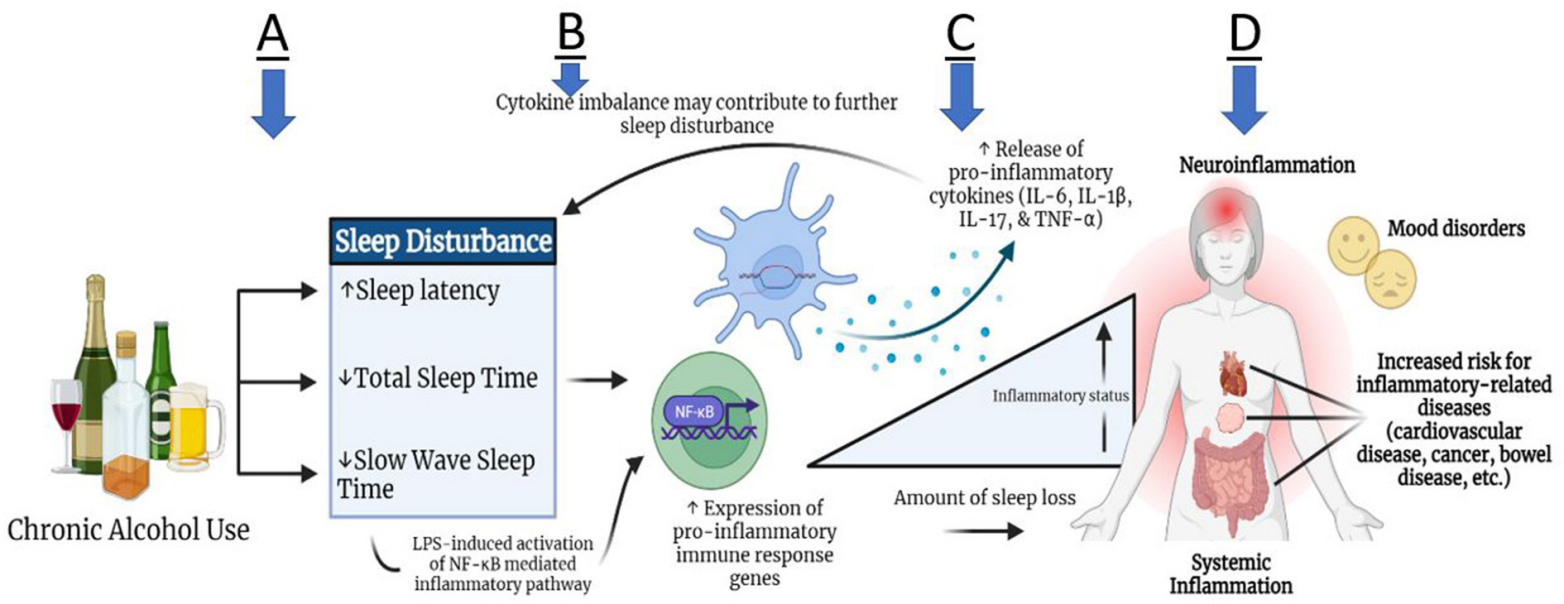
Theoretical relationship between alcohol use and sleep disturbance with inflammation. Subsequent to chronic alcohol use, some individuals may experience increased sleep latency, as well as decreased total sleep time and slow wave sleep time **(A)**. These sleep disturbances may contribute to heightened expression of pro-inflammatory immune response genes through the mechanism of toll-like receptor signaling, leading to leaked lipopolysaccharides (LPS) in plasma. LPS propagates the signal through different pathways, such as NF- κB, and induces cytokine production peripherally and in the brain **(B)**. Pro-inflammatory cytokine production (IL-6, IL-1β, IL-17, TNF-α) can lead to systemic inflammation and neuro-inflammation **(C,D)**. Due to the bidirectional link between the brain and periphery, the inflammatory activity can in turn lead to further altered sleep architecture. Ultimately, persistent inflammation can lead to an increased risk for inflammation-related comorbidities, such as cardiovascular disease, cancer, bowel diseases, and mood disorders **(D)**.

The specific effects of alcohol on an individual's sleeping patterns may depend on several factors such as, the amount and frequency of alcohol consumption, the time between consumption and bedtime, and the blood alcohol concentration (BAC) achieved near bedtime (8). Furthermore, alcohol can have either a stimulating effect that can increase sleep onset latency, or a depressant effect that can promote sleepiness (9). As alcohol metabolizes and blood alcohol levels fall, the second half of sleep may result in sleep fragmentation and longer periods of wakefulness. Chronic use of alcohol can severely disturb the normal stages of sleep, resulting in prolonged sleep latency in combination with observed deficits in both total sleep time (TST) and slow wave sleep (SWS) time (Figure 1A) (10).

Sleep disturbances can evoke a general inflammatory state by inducing the release of neurotransmitters such as norepinephrine, causing downstream cell signaling of inflammatory gene expression (11, 12). In addition to contributing to sleep disturbances, research has revealed chronic alcohol abuse to have metabolic consequences, namely cytokine expression imbalance (Figures 1B,C). Several studies have reported varying levels of Interleukin (IL)-1α, IL-1β, IL-6, IL-17, IL-18, monocyte chemoattractant protein-1 (MCP-1), and tumor necrosis factor-alpha (TNF-α) based on type of sleep condition and duration of sleep deprivation (13–19) (Figures 1B,C). van Leeuwen et al. (13) found that mRNA levels for IL-1β, IL-6, and IL-17 had increased significantly in 13 human participants after sleep restriction for five nights, whereas TNF-α mRNA levels did not change. Similarly, in a more recent pre-clinical study in mice, continuous sleep disruption for 3 days resulted in increased plasma cytokine levels such as IL-1β, IL-6 and TNF-α by 160.3, 51.9, and 213%, respectively (14). Other pre-clinical studies showed increased levels of IL-18 and IL-17 mRNA expression levels in the hippocampus of mice after acute rapid eye movement (REM) sleep deprivation for 3 days, highlighting the role of these cytokine expression levels in sleep modulation and inflammation pathways (15–17).

Cytokines MCP1, IL-17, HMGB1, IL-8, TNF-α, IL-1β, and IL-6 have been linked to chronic alcohol use in both preclinical studies in mice and in human patients in several studies (20–22). In fact, a recent study by Leggio et al. examined the acute effects of oral alcohol administration on four cytokines IL-6, IL-18, IL-10 and TNF-α among non-treatment seeking individuals with AUD. They found that plasma TNF-α levels were significantly reduced while IL-6 levels were increased 3 h following alcohol administration (23). Recently, a clinical emphasis has been placed on the cyclical effects of neuroimmune changes, brain physiology, and affective/behavioral outcomes in response to chronic alcohol use. Peripherally produced pro-inflammatory cytokines not only circulate systemically, but can also localize in multiple brain regions, alongside centrally produced cytokines from astrocytes or microglia cells (24, 25). A clinical study using human postmortem brains demonstrated increased MCP-1 concentration in the ventral tegmental area (VTA), substantia nigra (SN), hippocampus and amygdala of brains of individuals with AUD as compared with those without AUD (26). Thus, MCP-1 has been hypothesized to contribute to the neurodegenerative pathologies of chronic alcohol use. Continuous neuroinflammation from sustained drinking can manifest itself through cognitive and behavioral afflictions, such as fatigue, irritability, loss of concentration and appetite, and social withdrawal (Figure 1D) (27, 28). In the case of individuals with AUD, the withdrawal phase can induce disturbed sleep, depressive mood, and anxiety-like behavior, which can further exaggerate drinking habits (29–32).

Furthermore, alcohol use can alter liver function noted through changes in liver enzymes (Alanine transaminase-ALT, Aspartate transaminase-AST, Alkaline phosphatase-ALP, Gamma-glutamyltransferase-GGT), protein (Albumin and total protein) levels in blood (33–35) and markers of inflammation such as C-reactive protein (CRP) (36, 37). Another compound produced by liver during normal red blood cell breakdown, bilirubin, is seen at elevated levels in alcohol consumers (33).

Little is known about the relationship between sleep quality and cytokine levels within the AUD population. Therefore, we need to understand the mechanisms to manage the inflammatory pathways for the improvement of sleep quality, or possible role of sleep quality in regulation of pro-inflammatory cytokine levels in this population. The primary aim of this study was to explore the possible relationships between pro-inflammatory cytokine levels and the clinical biomarkers related to alcohol use such as liver and inflammation biomarkers, subjective measures of sleep quality, anxiety and depression disorders and differing drinking spectrums related to alcohol use.

## Methods

### Study population selection

This exploratory study included participants from a large Natural History of Alcohol Use protocol (NCT02231840) conducted by the National Institute on Alcohol Abuse and Alcoholism (NIAAA). The NCT002231840 protocol enrolled both treatment and non-treatment seeking individuals with AUD (meeting the DSM IV/5 criteria for AUD), as well as healthy volunteers. For the AUD case group, a total of 50 individuals from both treatment seeking (*n* = 25) and non-treatment seeking (*n* = 25) arm were included. The treatment-seeking patients with AUD were eligible to participate in other research protocols if they met the inclusion criteria. The subset of treatment seeking patients selected for this analysis were also participating in one of two ongoing experimental medicine trials; however only baseline plasma samples and data (prior to study medication intervention) were included in this analysis. Non-treatment-seeking AUD and healthy non-AUD participants were participating in acute alcohol administration studies, and only baseline plasma samples and data (prior to alcohol administration) were included in this analysis.

Participant selection for both the case and control groups in this secondary analysis was based on two criteria: participants must have had a stored plasma sample collected prior to enrollment into any experimental medication trial AND participants must have completed the sleep quality measure (Pittsburgh Sleep Quality Index, PSQI) at the time of enrollment. Race was categorized into the following three groups: African American/American Indian, White or Asian. Marital status was recoded into three groups as currently married/living with a partner, divorced or single.

### Study measures

Sleep quality measured by Pittsburgh Sleep Quality Index (PSQI) was assessed both on the continuous scale and as a dichotomous variable (38–42). When PSQI was dichotomized, individuals were categorized in the “poor” (≥5 PSQI) or “good” (<5 PSQI) sleep groups. Drinking history was assessed using the 90-day Timeline Followback (TLFB) measure (43, 44). The TLFB measure included: average drinks per day, number of drinking days, number of heavy drinking days and total drinks. The average drinks per day were calculated from the total drinks divided by number of drinking days and heavy drinking days were calculated as days with five or more drinks consumed for males and four or more drinks consumed for females across the 90-day period. Current state of anxiety and depression were assessed using two subscales of Comprehensive Pathological Rating Scale (CPRS); Brief Scale for Anxiety (BSA) and Montgomery Asberg Depression Rating Scale (MADRS) (45–47). Structured Clinical Interview for DSM-IV/5 Axis I Disorders (SCID) diagnosis of mood and anxiety were also used for this analysis (48, 49). Table 1 includes detailed description of all study measures and time of administration.

**Table 1:** Description and administration of study measures.

**Measure**	**Description**	**Timing of administration**
Pittsburgh sleep quality index (PSQI)	The Pittsburgh Sleep Quality Index (PSQI) is a 19-item, self-rated questionnaire used to measure sleep quality and disturbances over a one-month (30 days) time interval. Nineteen individual items generate seven “component” scores: subjective sleep quality, sleep latency, sleep duration, habitual sleep efficiency, sleep disturbances, use of sleeping medication, and daytime dysfunction. A global summation score of five or higher is indicative of poor sleep quality (38). The PSQI has been validated in populations with insomnia and other sleep disorders, with psychiatric patients, and in normal populations (39, 40). There exists some early evidence of validity in alcohol-dependent populations (41, 42). The PSQI has internal consistency and a reliability coefficient ranging from 0.80 to 0.83 for its seven components (38).	PSQI was administered on day 2 of inpatient stay for the treatment-seeking group and on the screening day for the non-treatment seeking and control group, as part of the natural history protocol.
Timeline follow-back (TLFB)	The TLFB collects drinking information using personal historical events recounted over a fixed time period (43). It is a standard assessment for measuring alcohol drinking patterns and quantification in treatment programs. The number of items corresponds to the number of days of interest, typically 90, which usually takes about 30 min to complete. The TLFB has demonstrated high test-retest reliability across multiple populations of drinkers. Content, criterion, and construct validity have been demonstrated in both clinical and general population samples (44). The study collected drinking data for 90 days preceding admission/screening	The TLFB was administered during the first week of inpatient stay for the treatment-seeking cohort and on the screening day for the non-treatment seeking and control group.
Comprehensive psychopathological rating scale (CPRS)	The CPRS is a 19-item measure that evaluates the present psychiatric state, along with the severity of symptoms and observed behaviors (45). CPRS includes two subscales: Brief Scale for Anxiety (BSA) and Montgomery Asberg Depression Rating Scale (MADRS) (46, 47). The BSA employs self-rated scales to gauge current symptoms of pathological anxiety alone or in combination of other psychological/medical disorders. The score for 10 items ranges from 0-60 with higher scores indicating higher anxiety. The MADRS subscale assesses core symptoms of depression and consists of 9 patient reported items and 1 based on interviewer's observation. The scores range from 0 to 6 for each item with total scores ranging from 0 to 60.	As part of the Natural History protocol, CPRS was administered on the screening day for the non-treatment seeking and control group, while weekly during the inpatient stay for the treatment seeking AUD group. For this analysis, only baseline values collected during week 1 are used.
Structured clinical interview for DSM-IV/5 Axis I disorders (SCID)	The SCID is an 11 module semi-structured interview used to make DSM-IV or 5 diagnoses (Diagnostic and Statistical Manual of Mental Disorders, version IV OR version 5). Trained professionals conduct interviews and final diagnoses are determined through a consensus process involving trained psychiatrists (48, 49)	SCID interviews were conducted for all participants at the time of enrollment as part of the Natural History protocol

### Plasma cytokine level profiling

Pro-inflammatory cytokine markers related to alcohol use were quantified using stored baseline plasma samples collected from all participants prior to enrollment into any drug interventional protocols. A total of 50 μL of frozen plasma samples were used for cytokine level assessment. The pre-defined Human inflammation panel 1 of the BioLegend^®^ LEGENDplex™ kit was used and all manufacturer's instructions were followed (50). The minimum detectable concentration (MDC) was defined as the theoretical limit of detection of the assay for any given cytokine using the LEGENDplex™ Data Analysis Software, by applying a 5-parameter curve-fitting algorithm. These values are different for each cytokine. The beads were analyzed by CytoFLEX (Beckman Coulter Life Sciences, Brea, CA) flow cytometer. The flow cytometry standard (FCS) files were submitted to and further analyzed by the LEGENDplex™ data analysis software (Biolegend). The following 13 cytokines were included in the pre-defined panel: Interleukin (IL)-1b, IL-8, IL-33, IFN-a2, IL-10, IL-23, IFN-g, IL-12p70, IL-18, TNF-α, IL-17A, IL-6, and MCP-1. The concentration of each cytokine was determined based on a known standard curve using the data analysis software provided by LEGENDplex™. All cytokine level values were expressed as pictograms per milliliter.

### Liver functions and inflammation markers

Because of the known possible effects of chronic alcohol use on liver functions, markers of liver function profile such as aspartate aminotransferase (AST), alanine aminotransaminase (ALT), alkaline phosphatase (ALP), gamma-glutamyl transferase (GGT), albumin, direct bilirubin, prothrombin time (PT), and partial thromboplastin Time (PTT) were included in this analysis. In addition, we explored C-reactive protein (CRP) as a general marker of inflammation. These data were extracted from the baseline clinical data for each participant before enrollment into any interventional trial. The hepatic panel and CRP test were done by NIH Clinical Center Department of Laboratory Medicine (DLM) on plasma samples, using the Abbott Architect immunoassay kit.

### Statistical analysis

#### Demographic and clinical variables assessment

Statistical analysis and data discovery were carried out using the JMP™ version 15 statistical software (SAS Headquarters, Cary, NC) and the IBM SPSS Statistics software. Graphical figures were generated in the JMP™ Statistical Discovery version 15 software. Student's or Welch's *t*-tests and Chi-square tests were used to assess differences in sleep quality (PSQI), CPRS anxiety (BSA) and Depression (MADRS) between the AUD group and the healthy controls. Pearson correlation was used to assess sleep measures (PSQI) and alcohol consumption variables within each group. To assess sex specific differences, PSQI measures and clinical diagnoses of mental health disorders including anxiety and mood disorders (SCID-IV/5) were compared between sexes in the AUD group only using a Student's *t*-test. Differences in liver and inflammation biomarkers values were assessed between the AUD population and the healthy controls using a Wilcoxon ranked sum test. Data are presented as mean ± standard deviation.

#### Cytokine level filtering and analysis

In order for a cytokine to be included in the univariate analyses, at least 40% of the samples must have met the minimal detectable concentration (MDC) limits of the assay (i.e., out of 64 samples assayed, at least 25 samples must have had a value at or above the MDC). After filtering, exploratory analyses were conducted to investigate associations between the cytokine plasma levels and specific demographic and clinical variables of interest. The explored variables of interest include sex, age, race, marital status, liver and inflammation profiles, drinking variables, mood and anxiety disorders and PSQI. Differences in cytokine levels were assessed between the AUD and healthy control groups using Wilcoxon signed rank test. Wilcoxon signed rank test (or Kruskal-Wallis if more than two groups) was used to assess differences in cytokine levels in sex (male, female), race (White, Asian, African American/American Indian), marital status (single, divorced, married) and between the presence or absence of mood and anxiety disorders based on SCID diagnosis and between “good” and “poor” sleepers based on PSQI. Within the AUD group alone, correlations between cytokine levels and liver and inflammation biomarkers, PSQI, CPRS anxiety and depression scores and drinking variables were assessed using Spearman correlation. Wilcoxon signed rank test was used to compare cytokine levels within the AUD on the following variables: good vs. poor sleepers, presence or absence of anxiety disorders and presence or absence of mood disorders.

#### Linear regression modeling

More rigorous filtering of cytokines was applied before running multiple linear regression models. In order for a cytokine to be included in the multiple linear regression models, all samples (*n* = 64) must have met the MDC requirements of the assay. Exploratory multiple linear regression models were run for cytokines to assess the relationships between those cytokine levels and the PSQI as well as differing drinking spectrums related to alcohol use, controlling for all other significant variables in the bivariate analyses. Age and the primary testing variables such as PSQI and significant drinking measurements were entered in the first block. Stepwise selection with entering criteria of *p* < 0.05 and removing criteria of *p* > 0.10 were used in the second block to select other variables in the final model. Normality and homoscedasticity were checked by residual and normal plot. Appropriate transformations were performed if necessary. Multicollinearity was checked by tolerance and variance inflation factor (VIF).

## Results

### Study population description

Average age was 39.5 ± 12.0 years for the AUD group and 25.4 ± 5.5 years for healthy controls (Table 2). With respect to the healthy controls, most of them were female (71.4%), white (71.4%) and single (100%), whereas among the AUD group, 51.9% were white females and 69.4% were single. Among the AUD group, 20 and 34% had SCID diagnosis of one or more mood and/or anxiety disorders, respectively, while none of the participants in the control group had any such diagnosis. Alcohol consumption assessed by TLFB showed that the AUD group had a mean average drinks per day of 8.5 ± 4.8 ranging from 2.5 to 22.5 drinks per day (Table 2). Healthy controls consumed on average 2.8 ± 1.0 drinks per day, ranging from 1.4 to 4.6 drinks per day.

**Table 2:** Demographic and clinical characteristics of study population (*n* = 64).

**Characteristic**	**AUD** **(*n* = 50)**	**Healthy controls** **(*n* = 14)**	***P*-value**
Age Mean ± SD (years)	39.5 ±12.0	25.4 ± 5.5	<0.001
	*N* (%)	*N* (%)	
Sex			0.195
Male	24 (48%)	4 (28.57%)	
Female	26 (52%)	10 (71.43%)	
**Race**			0.079
AA/AI	22(44%)	2 (14.29%)	
White	26 (52%)	10 (71.43%)	
Asian	2 (4%)	2 (14.29%)	
**Marital status**			0.073
Divorced	8 (16.33%)	0	
Married	7 (14.29%)	0	
Single	34 (69.39%)	14 (100%)	
Mood disorder (SCID IV/5)*			
Yes	10/50 (20%)	0	0.101
Anxiety disorder (SCID IV/5)*			
Yes	17/50 (34%)	0	0.013
	**Mean** **±SD**	
**CPRS**			
BSA	7.8 ± 8.1	1.0 ± 1.6	<0.001
MADRS	10.8 ± 10.8	1.5 ± 2.5	<0.001
PSQI Global Score	8.1 ± 4.4	2.4 ± 1.7	<0.001
**Drinking measures**			
Average drinks per day	8.5 ± 4.8	2.8 ± 1.0	<0.001
Number of heavy drinking days	54.7 ± 28.0	3.9 ± 4.6	<0.001
Number of drinking days	69.1 ± 21.1	21.6 ± 11.7	<0.001
**Liver and inflammation biomarker**			
ALP U/L	67.5 ± 19.0	58.6 ± 12.1	0.104
AST U/L	40.1 ± 52.3	19.0 ± 5.4	0.007
Albumin g/dL	4.3 ± 0.3	4.4 ± 0.2	0.258
Bilirubin direct mg/dL	0.08 ± 0.12	0.00 ± 0.00	<0.001
Bilirubin total mg/dL	0.48 ± 0.29	0.42 ± 0.12	0.23
GGT U/L	69.1 ± 82.5	14.6 ± 5.7	<0.001
PT-INR	0.97 ± 0.07	1.03 ± 0.05	0.01
PT-Auto seconds	13.18 ± 0.73	13.72 ± 0.53	0.011
PTT-Auto seconds	31.0 ± 3.1	32.9 ± 2.7	0.033
ALT U/L	40.1 ± 39.7	15.2 ± 11.1	<0.001
CRP mg/L	1.8 ± 2.2	0.7 ± 0.8	*0*.004

AA/AI, African American/American Indian; SCID, Structural Clinical Interview for DSM Disorders; CPRS, Clinical pathological rating scale; BSA, Brief scale of anxiety; MADRS, Montgomery-Asberg depression rating scale; TLFB, Time-line Followback; ALP, Alkaline Phosphatase; AST, Aspartate Aminotransferase; GGT, Gamma-glutamyl transferase; PT_INR, Prothrombin Time International Normalized Ratio; PT_Auto, Automated Prothrombin time; PTT_Auto, Automated Partial Thromboplastin Time; ALT, Alanine Transaminase; CRP, C-Reactive Protein. *SCID diagnosis of one or more disorder using the version IV or 5 (depending on the time of enrollment).

### Cytokine assay results

Plasma samples from 50 individuals with AUD (comprised of both treatment- and non-treatment seeking individuals) and 14 healthy volunteers were assayed to determine cytokine levels. Due to threshold constraints of the assay and certain samples not reaching the minimum detectable concentration (MDC), not all samples are included for any given cytokine. The total number of samples meeting the MDC for each cytokine as well as the aggregate statistics for both the raw and logarithm base 10 transformed cytokine level for each is provided in Supplementary Table S1. The MDC sensitivity levels for the assay are included in Supplementary Table S2. In order for a cytokine to be included in the analysis, at least 40% of the samples (25/64) must have met the MDC sensitivity level of the assay. The % of samples meeting the MDC is included in Supplementary Table S1. Of the 13 cytokines assayed, 7 met the filtering criteria and will be considered for the univariate analyses. The 7 cytokines and the corresponding percent of samples that met the MDC of each are as follows: TNF-a (52%), MCP-1 (100%), IL-8 (52%), IL-10 (40%), IL-18 (100%), IL-23 (42%) and IL-33 (76%). Two cytokines, MCP-1 and IL-18 had 100% of the samples at or above the MDC and were considered in the linear regression modeling.

### Sleep, drinking, mental health and liver profile variable assessment

**Figure 2:**
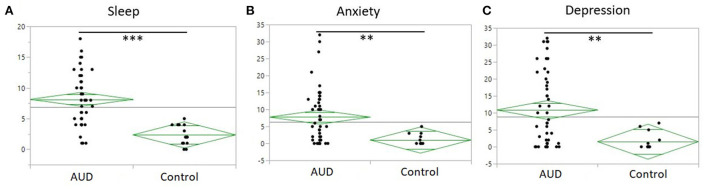
One-way plot of AUD and controls of sleep, anxiety and depression. **(A)** PSQI (y-axis) between AUD group and controls (x-axis). Significant difference of PSQI between AUD group and controls (*P* < 0.0001). Average PSQI in AUD group 8.14 to average PSQI in controls 2.35. **(B)** Anxiety measured by BSA (y-axis) between AUD group and controls (x-axis). Significant difference of anxiety in the AUD group (*P* = 0.003). **(C)** Depression measured by MADRS (y-axis) between AUD group and controls (x-axis). Significant difference of depression scores in AUD group (*P* = 0.002).

**Figure 3:**
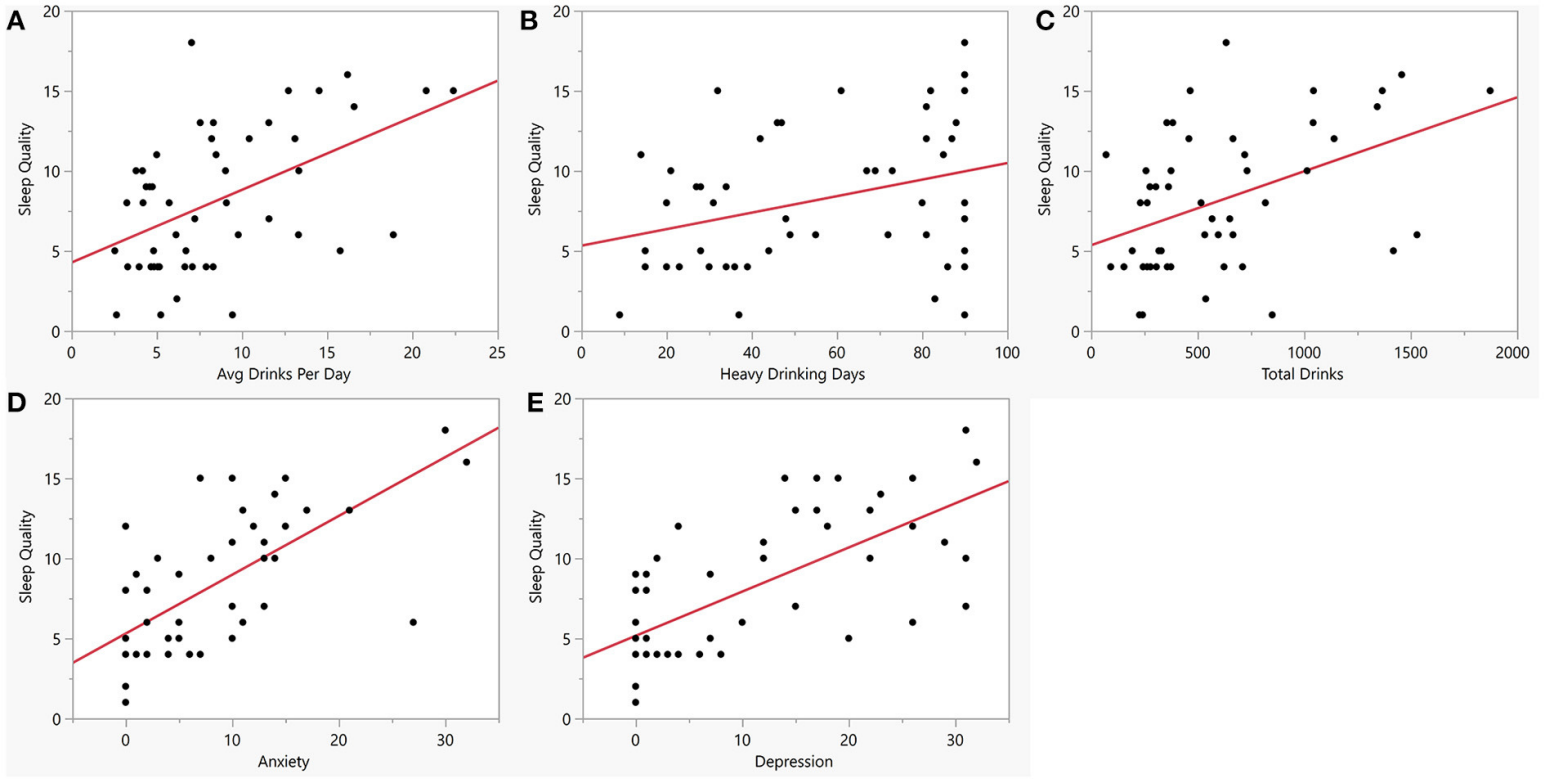
Bivariate plots between sleep quality, alcohol consumption, anxiety and depression within the AUD group. Y-axis is sleep quality (PSQI score) vs. **(A)** Average drinks per day (x-axis, AUD: R = 0.50, *p* < 0.001). **(B)** Number of heavy drinking days (x-axis, AUD: R = 0. 32, *p* = 0.02). **(C)** Total drinks (x-axis, AUD: R = 0.44, *p* = 0.001). **(D)** Anxiety measured by BSA (x-axis, AUD: R = 0.66, *p* < 0.001). **(E)** Depression measured by MADRS (x-axis, AUD: R = 0.67, *P* < 0.001). Red line represents regression line.

Sleep quality assessed as total PSQI score was significantly poorer among the AUD group (8.1 ± 4.3) as compared to controls (2.4 ± 1.7) (*p* < 0.001) (Figure 2A). Anxiety and depression scores were significantly higher among the AUD group compared to controls (*p*s < 0.001) (Figures 2B,C). Seven liver profile markers and C-reactive protein were found to be significantly different between the AUD group and controls including: CRP (*p* = 0.004), ALT (*p* < 0.001), Bilirubin direct (*p* < 0.001), GGT (*p* < 0.001), AST (*p* = 0.007), PT_INR, (*p* = 0.01), PT_Auto (*p* = 0.01), and PTT_Auto (*p* = 0.03) (Table 2). Within the AUD group, females (*n* =26, 9.7 ± 4.7) had significantly poorer sleep than males (*n* = 24, 6.5 ± 3.4) (*p* = 0.01; data not shown), higher scores for anxiety (10.0 ± 9.3) than males (5.4 ± 5.9) (*p* = 0.046) and reported higher levels of depression (14.4 ± 11.8) than males (7.1 ± 8.3) (*p* = 0.015). Interestingly, however, there were no significant differences between sexes in the TLFB drinking variables among the AUD group: average drinks per day, heavy drinking drinks days and number of drinking days. When sleep was examined for associations with drinking variables within each group, sleep quality had significant positive correlations (i.e., poorer sleep associated with higher alcohol consumption) with three TLFB drinking variables: average drinks per day (Figure 3A, *p* < 0.001), heavy drinking days (Figure 3B, *p* = 0.02) and total number of drinks (Figure 3C, *p* = 0.001) among the AUD group. The number of drinking days was not significantly correlated with sleep within the AUD group (*p* = 0.551). A significant positive correlation was also observed between sleep and anxiety (Figure 3D, *p* < 0.001) and depression (Figure 3E, *p* < 0.001) scores among the AUD group. Interestingly, there was also a significant positive correlation among the controls between sleep quality and both anxiety (*p* = 0.02) and depression (*p* = 0.02).

### Demographic variables and cytokine level associations

Across the entire study population (*N* = 64, both AUD and controls), MCP-1 (*p* < 0.001) was significantly positively correlated with age (data not shown). Within the AUD group, two cytokines were found to be significantly positively correlated with age: IL-23 (*p* = 0.022), and MCP-1 (*p* < 0.001) (Table 3). No cytokines were found to be significantly correlated with age within the healthy control group. None of the cytokines were found to be significantly different across sex, marital status, or race over the entire study sample nor within either group separately.

**Table 3:** Significant cytokine associations with age, sleep, anxiety, depression and drinking (AUD group).

					**Drinking (TLFB)**			
**Cytokines**	**Age**	**Sleep** **(PSQI)**	**Anxiety** **(BSA)**	**Depression** **(MADRS)**	**Avg drinks per day**	**Heavy drink days**	**No of drink days**	**Total drinks**
IL-8	−0.08	0.49*	0.45*	0.48*	0.41_*_	0.21	0.17	0.39*
IL-10	−0.05	0.33	0.51*	0.58**	0.45_*_	0.26	−0.12	0.41
MCP-1	0.54***	0.40**	0.39**	0.44**	0.51***	0.54****	0.42**	0.61****
TNF-a	0.21	0.24	0.16	0.25	0.29	0.43*	0.50**	0.46*
IL-23	0.50*	0.11	0.11	0.22	0.34	0.38	0.30	0.40

Numerical values represent Spearman Correlation Coefficient. Asterisk indicates significance level (The *, **, ***, and **** symbols indicate the value of P < 0.05, P < 0.01, P < 0.001, and P < 0.0001 respectively).

### Clinical variables and cytokine level associations

**Figure 4:**
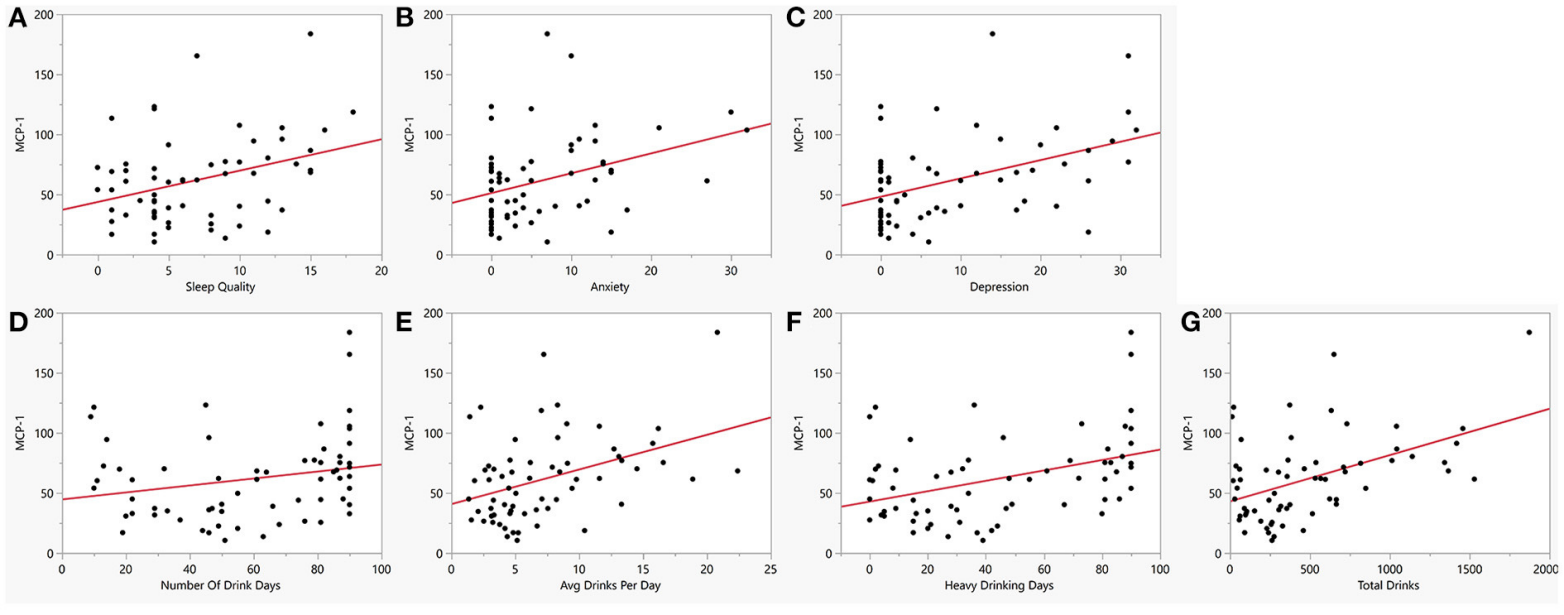
Bivariate plot of cytokine MCP1 and co-occurring symptoms in AUD. MCP-1 (y-axis) vs. **(A)** Sleep measured by PSQI (x-axis, *R* = 0.40, *p* = 0.004), **(B)** Anxiety measured by BSA (*x*-axis, *R* = 0.39, *p* = 0.006), **(C)** Depression measured by MADRS (x-axis, *R* = 0.44, *p* = 0.002), **(D)** Number of drinking days (x-axis, *R* = 0.42, *p* = 0.002), **(E)** Average drinks per day (x-axis, *R* = 0.51, *p* < 0.001), **(F)** Heavy drinking days (x-axis, *R* = 0.54, *p* < 0.001), and **(G)** Total number of drinks (x-axis, *R* = 0.61, *p* < 0.001). Red line represents regression line.

Cytokine level associations within the case and control groups were assessed separately among anxiety, depression, sleep and the following four drinking variables: average drinks per day, number of heavy drinking days, number of drinking days and the total drinks. Within the AUD group, two cytokines, IL-8and MCP-1 were found to be positively correlated with sleep, anxiety, depression and at least two of the four drinking variables (Table 3). In particular, MCP-1 was significantly positively correlated with all seven variables. Higher levels of MCP-1 were associated with poorer sleep (Figure 4A, *p* = 0.004), higher scores of anxiety (Figure 4B, *p* = 0.006) and higher scores of depression (Figure 4C, *p* < 0.002). Furthermore, individuals with AUD who had high consumption levels such as number of drinking days (Figure 4D, *p* = 0.002), high average drinks per day (Figure 4E, *p* < 0.001), high number of heavy drinking days (Figure 4F, *p* < 0.001) and high total number of drinks (Figure 4G, *p* < 0.001) all had significantly higher levels of MCP-1. Another cytokine IL-8 was found to be significantly correlated with sleep, anxiety, depression, average drinks per day and the total number of drinks (Supplementary Figure S1A) among the AUD case group. The cytokine IL-10 (Supplementary Figure S1B) was found to be significantly correlated with anxiety, depression and average drinks per day but not sleep or the other three drinking variables. In addition to this, TNF-α was found to be significantly associated with three of the four drinking variables: heavy drinking days (*p* = 0.03), number of drinking days (*p* = 0.01) and total drinks (*p* = 0.02). Finally, when controls were assessed, only MCP-1 was found to be associated with the number of drinking days (*p* = 0.002) and total drinks (*p* = 0.02) but not with average drinks per day or number of heavy drinking days.

**Figure 5:**
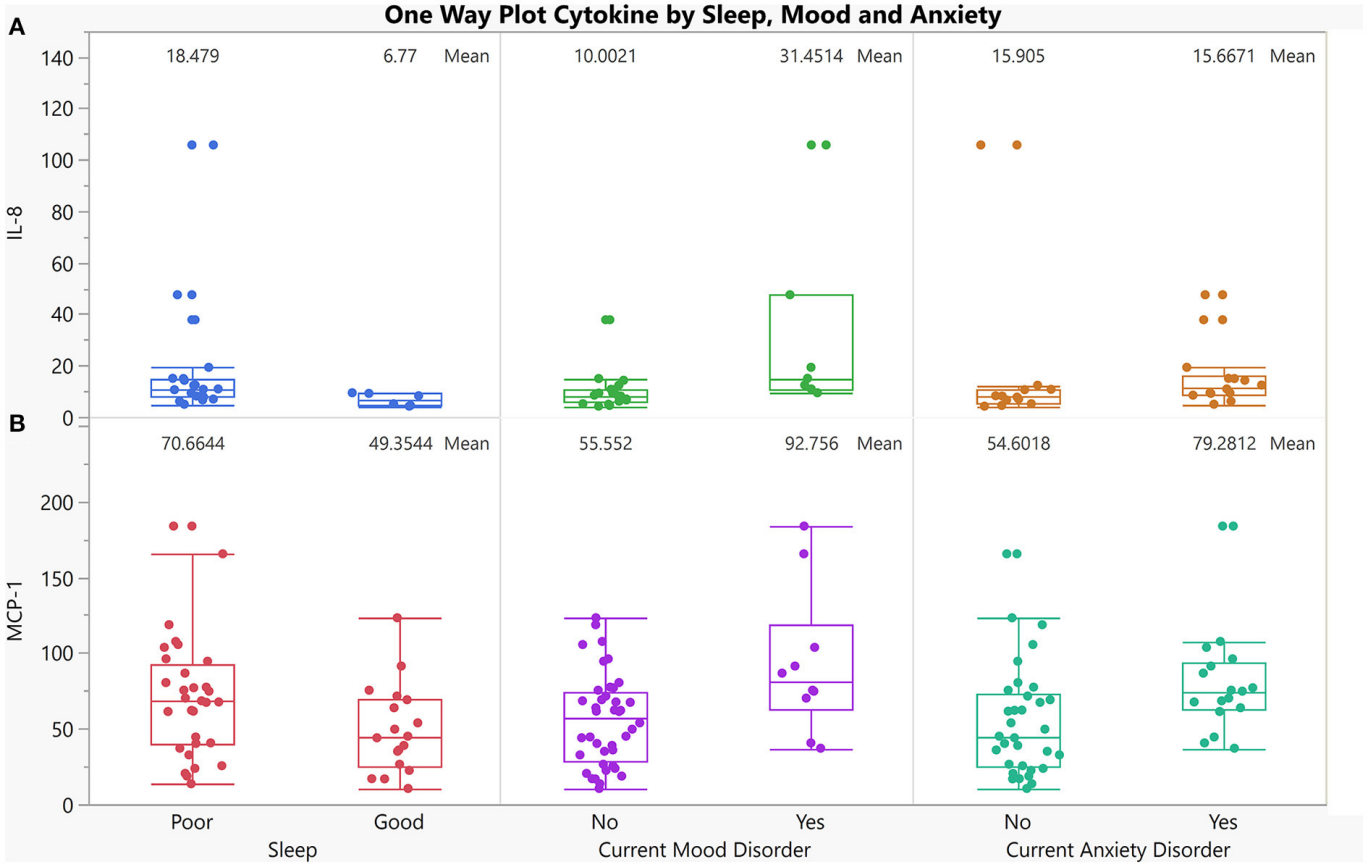
One-way plot of IL-8, and MCP-1 vs. co-occurring dichotomized symptoms in AUD. X-axis indicates 3 dichotomized symptoms in individuals with AUD who reported having good sleep and those who reported having poor sleep (first panel), individuals with AUD who currently have a mood disorder compared to those who currently do not have a mood disorder (2nd panel), individuals with AUD who currently have an anxiety Disorder compared to those who currently do not have an anxiety disorder (3rd panel). Y-axis is cytokine levels of: **(A)** IL-8 (*p* = 0.02, *p* = 0.003, *p* = 0.04 respectively for sleep, mood and anxiety), **(B)** MCP-1 levels (*p* = 0.05, *p* = 0.01, *p* = 0.01 respectively for sleep, mood and anxiety).

To better understand these relationships, cytokine levels were assessed between presence/ absence of anxiety (Yes, 17/50), presence/absence of mood disorders (Yes, 10/50) and between good/poor sleepers (Poor, 32/50). Two cytokines, MCP-1, and IL-8 showed significantly different levels with at least two of the three dichotomized grouped variables (Figure 5). IL-8 and MCP-1 were found to be significantly higher in AUD individuals with the presence of a mood (*p* = 0.003, *p* = 0.01, respectively) and anxiety (*p* = 0.04, *p* = 0.01, respectively) disorder and those who have poor sleep (*p* = 0.02, *p* = 0.04) (Figures 5A,B).

### Correlations between liver biomarkers and cytokine levels

**Figure 6:**
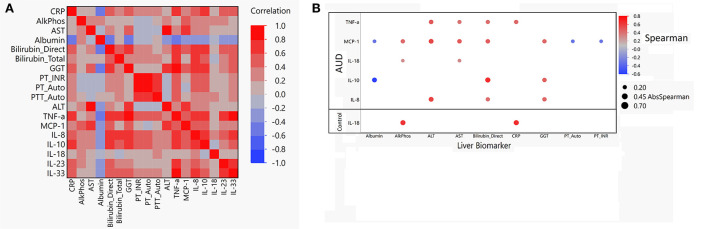
Spearman correlation matrix and dot plot of cytokines compared to liver function biomarkers. **(A)** Spearman correlation heat map of cytokines vs. liver biomarkers in AUD. Red indicates a Spearman correlation of 1 and blue indicates a Spearman correlation of −1. **(B)** Dot map of significant correlation of cytokine compared to liver biomarker for both AUD and controls. Blue dot indicates negative correlation and red dot indicates positive correlation. Size of the dot indicates absolute value of the magnitude of the correlation. If dot is present, *P* < 0.05 was reached.

Multiple significant correlations were found between cytokine levels and liver profile markers within the AUD group (Figure 6A). Within the AUD group, 5/7 cytokines were found to be significantly correlated with at least one of the liver profile biomarkers (Supplementary Table S3; Figure 6B). Of the 5 cytokines found to be significantly correlated with the liver profile biomarkers, MCP-1, and IL-10 were significantly negatively correlated with Albumin. Cytokine MCP-1 was also significantly negatively correlated with PT-Auto and PT-INR. All other included cytokines were significantly positively correlated with ALP, ALT, AST, Bilirubin direct, CRP, and GGT. When the control group was assessed, cytokine IL-18 was significantly positively correlated with ALP and CRP (Supplementary Table S3; Figure 6B).

### Multiple linear regression model results

MCP-1 and IL-18 showed levels above the MDC for all 50 AUD samples assayed, and therefore passed the requirement of <10% of missing values. Given this, these two cytokines were investigated using multiple linear regression modeling. Multicollinearity was checked where tolerance and variance inflation factors (VIF) ranged from 0.577 to 0.907 in MCP-1, indicating no multicollinearity concerns among any of the predictor variables. The final model accounted for 56.3% of the variability in MCP-1 (Table 4). Controlling for sleep status and heavy drinking days, participants who were older (*p* = 0.003), had more drinks per day (*p* = 0.016), and had higher ALP level (*p* = 0.001) reported higher MCP-1 levels. The final IL-18 model with age, PSQI, heavy drinking days, and average drinks per day was not significant (data not shown, *p* = 0.93).

**Table 4:** Multiple linear regression results MCP-1.

**Variable**	**Beta (std. error)**	***p*-value**
Age	0.008 (0.003)	**0.003**
Average drinks per day	0.019 (0.008)	**0.016**
Heavy drinking Days	0.001 (0.001)	0.285
PSQI score	−0.031 (0.066)	0.644
Alkaline phosphatase levels	0.005 (0.002)	**0.001**

Significant p-values are bolded.

## Discussion

This study was aimed at investigating associations between pro-inflammatory cytokine levels, sleep quality, and anxiety/mood disorders in individuals with AUD. Considering the known relationship between heavy alcohol use and sleep disturbances and the existence of mood and anxiety disorders in individuals with AUD (Figure 1) (5, 7, 10, 30), higher levels of sleep disturbance (Figure 2A), anxiety and depression (Figures 2B,C) was observed in individuals with AUD compared to the healthy volunteers. Furthermore, not surprisingly, the individuals with AUD reported poorer sleep quality in relation to the Timeline Followback (TLFB) variables such as higher average drink consumption per day, number of heavy drinking days and higher total drinks (Figures 3A–C). Additionally, poorer sleep quality was significantly correlated with higher anxiety and depression scores (Figures 3D,E). Two cytokines showed significant associations among almost all the co-occurring symptoms present in individuals with AUD. Specifically, inflammation as measured by higher cytokines levels of IL-8, and MCP-1 were found to have positive significant associations with sleep, anxiety, depression and TLFB assessed drinking outcomes. Specifically, cytokine MCP-1 was found to be positively correlated with poor sleep, high anxiety and depression and higher drinking measures (Figure 4).

Among the AUD group, many of the standard inflammatory liver biomarkers were positively correlated with 5 cytokines except albumin, a standard measure of liver and kidney function, which was negatively correlated with cytokines MCP-1, and IL-10. Albumin was also negatively correlated with alcohol consumption in the AUD group indicating that heavier alcohol use leads to lower albumin levels, a commonly understood symptom of heavy alcohol use. These exploratory findings may help to further understand the prolonged inflammatory response and higher risk of chronic disease related to heavy alcohol use.

Sleep and alcohol use have a bi-directional relationship; that is, those with existing sleep conditions may self-medicate using alcohol given its sedating properties (51, 52), and heavy alcohol use can also negatively impact sleep quality (53, 54). Because of this, poor sleep quality could be a potential risk for the development as well as relapse of AUD (55). Although our study was not powered to assess causality, consistent with prior reports, individuals with AUD in this secondary analysis reported significantly lower sleep quality (indicate by higher PSQI global scores) as compared to healthy controls. Given the associations between both subjective and objective sleep measures and relapse among individuals with AUD (56, 57), more focused research is needed to inform the pathways through which these relationships occur.

Chronic alcohol use and sleep disturbances can cause higher levels of circulating cytokines with increased risk for inflammatory disease (11, 21, 22), however, detecting these effects may require a large longitudinal study. In our study with a small sample size, we implemented a multiple linear regression model on two cytokines that had 100% of the samples assayed and found that after controlling for the sleep (PSQI) and heavy drinking days (assessed by TLFB), older individuals with higher alkaline phosphatase levels and consuming higher average drinks per day had higher levels of MCP-1 (age *p* = 0.020, ALP *p* = 0.009). Our results also demonstrate significant associations between some cytokine levels and drinking history; specifically, MCP1 and IL-18 levels were significantly higher with different drinking history outcomes confirming previous reports of changes in the inflammatory response with chronic use of alcohol (58). Leggio et al. specifically studied levels of four pro-inflammatory cytokines (TNF-α, IL-10, IL-18 and IL-6) in a group of 25 non-treatment seeking individuals with AUD at baseline and following oral alcohol administration. They found that after 3 h of oral alcohol administration, there was a significant reduction in plasma levels of TNF-α while significant increase in IL-6 levels (23). While these findings cannot be directly compared to ours since we do not have a longitudinal design, this study and these findings show an acute effect that alcohol might have on inflammation markers in the plasma. Subsequently, in a pre-clinical study, Lowe and colleagues reported alcohol induced alterations in pro-inflammatory expression levels in brain and intestines, specifically significantly higher expression of MCP-1, TNF-α, Hmgb1, IL-17, and IL-23 (22). Our results also demonstrate significant associations between sleep quality and the following three cytokines, IL8, IL10, and MCP-1 among individuals with AUD. These findings confirm the previous reports of altered pro-inflammatory cytokine levels with sleep disturbance (11, 18). With chronic sleep disturbance among individuals with alcohol use disorder, these elevated cytokine levels may pose increased inflammatory disease risk and associated morbidity and mortality.

Due to the strong evidence of co-occurrence of alcohol use and mental health disorders (59), we collected both patient-reported measures and clinical diagnosis of anxiety and depression. In our study population, most of the individuals with AUD had one or more mood and/or anxiety disorder whereas none of the individuals in control group had a diagnosis of mood or anxiety. Furthermore, the control group also had very low scores on patient-reported measures of anxiety and depression. Previously, Klimkiewicz et al. (59) reported in their review of literature that more than one third (37%) of the individuals with alcohol dependence had a comorbid psychiatric disorder such as mood, anxiety and/or depression, whereas Brooks et al. (60) reported higher sleep regularity scores among treatment seeking individuals with AUD who were not diagnosed with any mood disorder. We also found significant associations between mood and anxiety and pro-inflammatory cytokine levels (MCP-1 & IL8) among individuals with AUD. These increased cytokine levels in chronic alcohol use, especially MCP-1, may in turn facilitate the neurodegenerative changes contributing toward even higher psychological conditions (26). Therefore, altering these inflammatory pathways could be a viable strategy for targeting these co-morbid conditions such as mood, anxiety and sleep disturbances. A major point to note here is that the subjective reports of anxiety and depression in our analysis were validated by clinical diagnosis of these psychiatric disorders, meaning that we found almost similar associations between these cytokines and CPRS mood/anxiety scores and SCID diagnoses of mood and anxiety.

We investigated the TLFB drinking variables between males and females within each group and observed no significant differences. This is contrary to what is reported in literature stating that males consume more alcohol than females and males experience more adverse events related to alcohol use. Interestingly, a report in 2020 indicated that these alcohol consumption gaps between the sexes is narrowing (61). Within the AUD group specifically, males reported having slightly more drinking days than females during the previous 90 days but the difference was not significant (*p* = 0.30). Furthermore, females reported drinking more average drinks per day than males in this study sample but this difference was not significant (*p* = 0.44). In order to confirm these findings, a larger sample size would need to be investigated since this study is comprised of only 24 males and 26 females with AUD.

Alcohol is metabolized primarily through liver, thus making it a major target for ethanol toxicity (62). Our results show significant differences in several liver markers between the two groups, potentially indicating liver biomarker disruption from heavy alcohol use. Alcohol induced activation of innate immunity in liver triggers activation (increased production of pro-inflammatory mediators) of inflammatory pathways (63). To this note, we found significant associations between most of the liver enzymes (ALT, AST, GGT, ALP) and pro-inflammatory cytokine levels (IL10, & MCP-1) within the AUD group. These findings support the current evidence that with chronic alcohol use, barrier function of the intestinal mucosa is impaired leading to increased lipopolysaccharide (LPS) to the portal circulation (62). This in turn activates innate immunity resulting in increased levels of pro-inflammatory cytokines such as MCP-1. Mandrekar et al. (64) found in their *in vitro* experiment, that there was an enhanced production of TNF-α cells when monocytes were exposed to alcohol for four or more days (63). Our findings also highlight significant negative association between plasma albumin levels and drinking variables as well as two cytokine levels. To understand the exact relationships between chronic alcohol use and inflammatory responses, larger studies with longitudinal designs are required, since interventions targeting the immune system could be a potential approach in developing novel treatments for alcohol induced liver damage/morbidities.

While this study highlights important associations between inflammation, sleep and heavy alcohol use, there are limitations that must be noted. The sample population of this exploratory analysis was not specifically designed to study pro-inflammatory cytokine levels with relation to disrupted sleep in AUD since samples were compiled from a large Natural History of Alcohol Use protocol conducted by the NIAAA. The plasma sample collection from the AUD group was not conducted simultaneously before any intervention nor was it collected at approximately the same time frame as the collection of the sleep and other study measures. That said, all plasma samples for cytokines, samples for liver function markers and self-reported measures were obtained within the first 2 weeks of inpatient treatment for the treatment-seeking individuals. The control group was significantly younger than the case group thus, it is important to mention that this difference could have confounded some of the inferences made on cytokine level differences between the AUD group and controls regardless of drinking. While the control group were used as a healthy group for comparison, it is important to note that they were not entirely abstinent from alcohol. All individuals within the control group did consume some alcohol and had an average drinks per day range of 1.3 to 4.5 (mean: 2.7 drinks per day). However, there was only one individual in the control group who reported having more than 10 heavy drinking days (16 total) in the TLFB. That said, the remaining 13 healthy volunteer individuals reported <10 heavy drinking days over the 90 days report of the TLFB. While the healthy control group did report having some alcohol use, it is important to note that none of the individuals in the healthy control group had comorbid mood disorders whether they reported consuming alcohol or not.

Although we found significant associations between cytokine levels, alcohol use within the last 90 days and sleep, we were unable to control other factors contributing to those associations, such as recent alcohol use, diet, co-morbidities, and medication use. The LEGENDplex™ (BioLegend^®^) kit used for the cytokine assays was a multiplex system that enabled us to look at a large number of pro-inflammatory cytokines panel. However, a limitation was the proportion of missing data where many of the samples included were not able to reach the MDC of the assay. Once cytokines were filtered, only 7 of the 13 cytokines were considered for any analyses, therefore reducing the number of tests in the study. Since this data reduction step removed almost half of the cytokines considered, no multiple comparison correction was applied to the smaller dataset. Furthermore, due to this limitation, complex regression modeling across all cytokines and other markers could not be conducted. Future studies investigating this concept should focus on designing larger case/control groups with age/gender matched samples with a larger panel of pro-inflammatory cytokines. Future studies should also take into account the detection limits of the assay being used. A longitudinal design could help to further understand the changes in cytokine levels based on alterations in alcohol consumption and sleep quality. Of note, however, is that a major strength of this analysis was that both groups had many study measures collected that could be investigated with cytokine levels before any intervention was done.

## Conclusion

This exploratory analysis suggests the need to further study the relationship between markers of inflammation and sleep among treatment seeking individuals with alcohol use disorder. A clear understanding of the dynamic relationship between sleep disturbance and immune pathways in treatment seeking individuals with AUD may enable clinicians to develop, evaluate and recommend focused therapeutic strategies to support individuals with AUD from treatment through recovery to maintain abstinence and improve sleep quality.

## Data availability statement

Aggregate cytokine level values are available as Supplemental Table 1. Clinical data used in this study are not publicly available due to ethical concerns regarding patient privacy and original patient consent. Data may be made available by requests directly to the corresponding authors.

## Ethics statement

Ethical review and approval was obtained in accordance with local legislation and institutional requirements for the clinical studies from which data for this publication were obtained. The participants provided their written informed consent to participate in this study.

## Author contributions

GW, ND, MS, and VR were the PIs of the parent protocols used for this secondary analysis. GW and VR provided funding and research support. BG and DF provided lab support, ran the assays, and generated cytokine data. NK, GW, and VR developed the concept. NK, GW, JB, and VR designed the analytical approach. JB and LY conducted the data analysis. NK, GW, JA, LY, JB, and VR interpreted the findings and drafted the manuscript. All authors provided critical feedback and approved the final version for publication.

## Funding

This research was supported by intramural research funds from the National Institutes of Health, Clinical Center and National Institute on Alcohol Abuse and Alcoholism.

## Conflict of interest

The authors declare that the research was conducted in the absence of any commercial or financial relationships that could be construed as a potential conflict of interest.

## Publisher's note

All claims expressed in this article are solely those of the authors and do not necessarily represent those of their affiliated organizations, or those of the publisher, the editors and the reviewers. Any product that may be evaluated in this article, or claim that may be made by its manufacturer, is not guaranteed or endorsed by the publisher.

## Abbreviations

IL: Interleukin
MCP: Monocyte Chemoattractant Protein-1
IFN-g: Interferon Gamma
ALT: Alanine transaminase
AST: Aspartate Aminotransferase
GGT: Gamma-glutamyltransferase
ALP: Alkaline phosphatase
CRP: C-reactive protein
AUD: Alcohol Use Disorder
SCID: Structured Clinical Interview for DSM-IV/5 Axis I Disorders
TLFB: Timeline Followback
PSQI: Pittsburgh Sleep Quality Index
REM: Rapid Eye Movement Sleep
CPRS: Clinical pathological rating scale
BSA: Brief Scale of Anxiety
MADRS: Montgomery Asberg Depression Rating Scale.
