# Supplementary material for: An exploratory study of pro-inflammatory cytokines in individuals with alcohol use disorder: MCP-1 and IL-8 associated with alcohol consumption, sleep quality, anxiety, depression, and liver biomarkers
Source: Front Psychiatry. 2022 Aug 11;13:931280. doi: 10.3389/fpsyt.2022.931280 (PMC9405018; doi:10.3389/fpsyt.2022.931280)
Supplement: Supplementary file 1 [file Data_Sheet_1.docx]

**Supplemental Figure and Tables**

**Supplemental Figure 1**


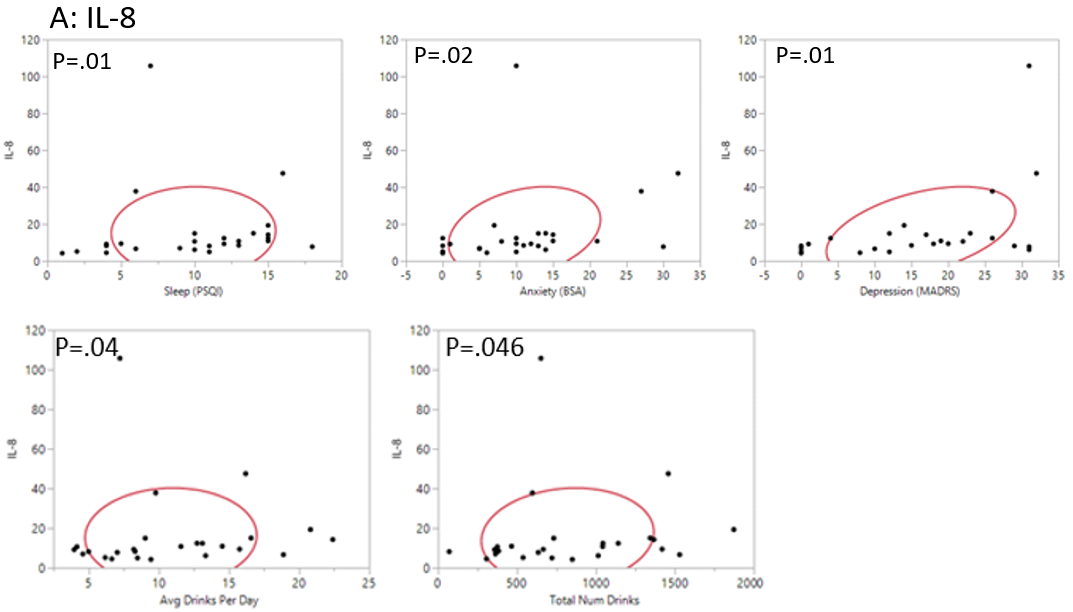


B. IL-10


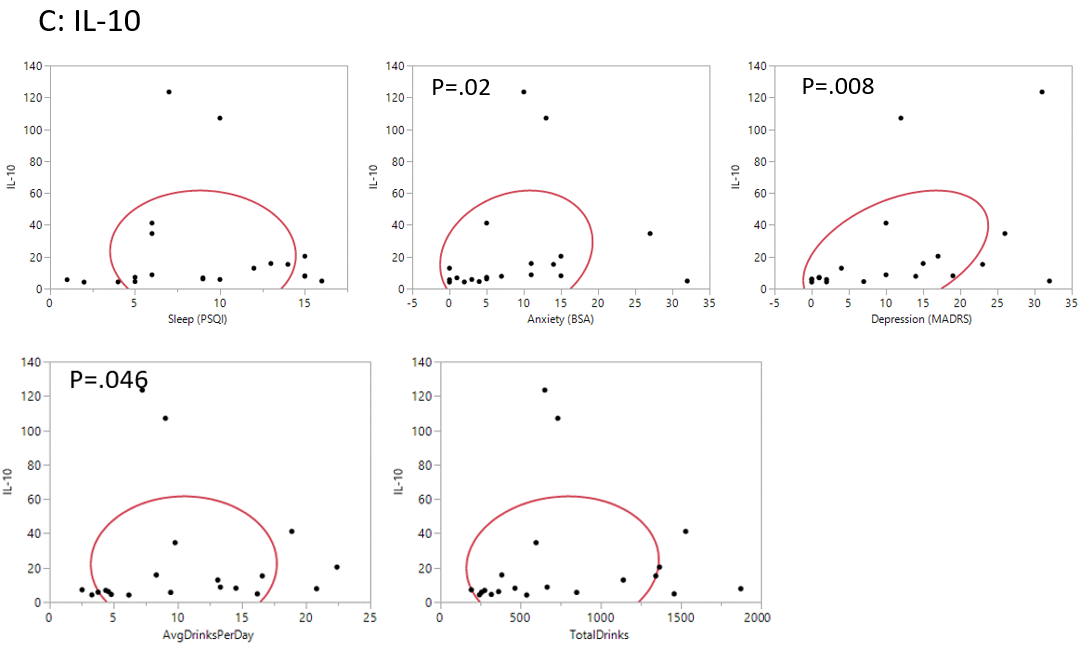


Legend: Bivariate plots of cytokine level (y-axis) A)IL-8, B)IL-10 versus co-occurring symptoms in AUD (x-axis): Sleep, Anxiety, Depression, Average Drinks per day, Total number of drinks. When a significant correlation is observed (Spearman), P<.05 is indicated on the plot.

**Supplemental Table 1: Supplemental_Table1_Cytokine_Descriptives.xls**

**Supplemental Table 2: Sensitivity levels of cytokine analyte of the assay.**The minimum detectable concentration (MDC) is the theoretical limit of detection calculated using the LEGENDplexTM Data Analysis Software by applying a 5-parameter curve fitting algorithm. Assay sensitivity presented here is MDC + 2x STDEV.

| **Analyte** | **Sensitivity in Serum (pg/mL)** |
| --- | --- |
| **MCP-1** | 1.1 + 1.2 |
| **IFN-alpha** | 2.1 + 0.2 |
| **IFN-gamma** | 1.3 + 1.0 |
| **IL-1beta** | 1.5 + 0.6 |
| **IL-6** | 1.5 + 0.7 |
| **IL-8** | 2.0 + 0.5 |
| **IL-10** | 2.0 + 0.5 |
| **IL-12 p70** | 2.0 + 0.2 |
| **IL-17A** | 0.5 + 0.1 |
| **IL-18** | 1.3 + 0.9 |
| **IL-23** | 1.8 + 0.1 |
| **IL-33** | 4.4 + 1.5 |
| **TNF-alpha** | 0.9 + 0.8 |

**Supplemental Table 3: Correlations between liver biomarker variables and cytokine levels.**

| **Group Significant Correlation** | **Liver Marker** | **Cytokine** | ***Prob>\|p\|*** | **Spearman** |
| --- | --- | --- | --- | --- |
| Alcohol Use Disorder | Albumin | IL10 MCP-1 | .007 .031 | -.5822  -.3054 |
|  | Alkaline Phosphatase | IL18 MCP-1 | .042  .001 | .2886  .4452 |
|  | ALT | TNF-a  MCP-1  IL-8 | .017  <.001  .002 | .4965  .5668  .5847 |
|  | AST | TNF-a  MCP-1  IL-18 | .012  <.001  .028 | .3109  .3946 |
|  | Bilirubin_Direct | TNF-a  IL-8  MCP-1  IL-10 | .032  .029  <.001  .001 | .4208  .4267  .4812  .6635 |
|  | C-Reactive Protein | TNF-a | .023 | .4441 |
|  | GGT | MCP-1  IL-8  IL-10 | .0013  .019  .035 | .4429  .4572  .4737 |
|  | PT_Auto/PT_INR | MCP-1  / MCP-1 | .029  /  .037 | -.3091  /  -.2962 |
|  |  | | | |
| Control | Alkaline Phosphatase | IL-18 | .013 | .6425 |
|  | C-reactive protein | IL-18 | .009 | .6646 |
